# Successful Treatment of Crizotinib-Induced Fulminant Liver Failure: A Case Report and Review of Literature

**DOI:** 10.1155/2020/8247960

**Published:** 2020-03-10

**Authors:** Kyle Kreitman, Satheesh P. Nair, Jiten P. Kothadia

**Affiliations:** MUH James D. Eason Transplant Institute, University of Tennessee Health Sciences Center, 1265 Union Avenue, 4 Shorb Tower, Memphis, TN 38104, USA

## Abstract

Crizotinib is a first-line tyrosine kinase inhibitor used for the treatment of metastatic lung cancer. Crizotinib-induced hepatotoxicity is a rare event. We report a case of a 46-year-old female with a history of metastatic lung cancer who presented with acute liver failure after being on crizotinib for two months. The medication was discontinued, and she was treated with N-acetylcysteine for seven days. Her liver function tests returned to normal limits after 26 days after admission. The precise mechanism and risk factors of crizotinib-induced hepatotoxicity remain unknown. Physicians should be aware of the potentially lethal side effect caused by crizotinib.

## 1. Introduction

Lung cancer is the second most common cancer in both men and women. It is the leading cause of cancer death, with about 142670 deaths a year in the United States [1]. There are approximately 1.3 million new patients diagnosed worldwide each year. Non-small-cell lung cancer (NSCLC) accounts for 85% of lung cancer patients. Of those diagnosed with NSCLC, 4–7% are positive for the anaplastic lymphoma kinase (ALK) gene rearrangement [2]. Targeting the ALK gene rearrangement has been shown to be more effective in patients with advanced NSCLC when compared to standard chemotherapy, and it is the first-line therapy for these patients [3].

Crizotinib is a tyrosine kinase inhibitor that targets ALK, mesenchymal epithelial transition factor (MET), and ROS1 proto-oncogene receptor tyrosine kinase (ROS1) [4]. The FDA has approved crizotinib as the first-line agent for the treatment of ALK-positive NSCLC. Common side effects of crizotinib include vision changes, fatigue, anorexia, diarrhea, nausea, vomiting, fatigue, dizziness, and upper respiratory tract infection [5, 6]. Elevated liver function tests have been reported with crizotinib treatment, which usually resolves with cessation of the drug or dose adjustment. Although rare, crizotinib-induced severe hepatotoxicity with fatal outcome occurred in 0.1% of patients in clinical trials [5]. Review of the literature extracted 12 cases of crizotinib-induced liver injury, with 66% (8/12) mortality. We report a case of crizotinib-induced fulminant liver failure which was treated with discontinuation of the drug and N-acetylcysteine with complete recovery.

## 2. Case Report

A 46-year-old African American female with a past medical history significant for metastatic NSCLC, ovarian vein thrombosis, and gastroesophageal reflux disease (GERD) presented to our hospital with a primary complaint of intermittent right upper quadrant pain over a two-week duration. Her symptoms were associated with nausea, generalized weakness, jaundice, dark urine, and mild dyspnea on exertion. The patient denied any history of alcohol use, smoking, illicit drug use, family history of liver disease, or history of recent travel outside of the United States. She was not sexually active.

Regarding her history of lung cancer, she was diagnosed to have stage IV metastatic adenocarcinoma of the lung (T2aN3M1c, Stage IVB) approximately nine months ago. At the time of diagnosis, she was noted to have intracranial, skeletal, and lung metastases along with generalized lymphadenopathy. The patient was started on chemotherapy regimen with carboplatin and paclitaxel in March 2019. The chemotherapy was subsequently discontinued as the molecular analysis of the lung cancer specimen showed a ROS1 gene rearrangement. She was started on treatment with crizotinib 250 mg twice daily (PO). Four weeks after initiation of crizotinib, her transaminases were noted to be elevated and treatment was discontinued. Few weeks after discontinuation of crizotinib, her liver function tests returned to normal. She was restarted on crizotinib at a lower dose of 200 mg twice daily with close monitoring of liver function tests every 2 weeks. On day 14, her AST was 60 U/L (15–37) and ALT was 55 U/L (16–61) with normal total bilirubin and alkaline phosphatase, and crizotinib was continued. However, 4 weeks after reinitiation of crizotinib, she presented to the emergency room with jaundice.

On physical exam, she had scleral icterus and right upper quadrant tenderness but without guarding, rebound, or rigidity. She was alert and oriented, without any focal deficits and asterixis.

Liver function tests at the time of admission showed AST 4093 U/L (15–37), ALT 3653 U/L (16–61), total bilirubin 5.8 mg/dL (0.2–1), conjugated bilirubin 4.5 mg/dL, and alkaline phosphatase 358 U/L (45–117). International normalized ratio (INR) was 10 (0.8–1). The white blood cell count was 4.0 × 10^3^/microL (4.2–10.2). The platelet count was 349 × 10^3^/microL (150–400). Her model for end-stage liver disease (MELD) score was 39. Serologic studies for hepatitis A, hepatitis B, hepatitis C, hepatitis E, human immunodeficiency virus (HIV), treponema pallidum, cytomegalovirus, Epstein–Barr virus, herpes simplex virus, and parvovirus were negative. Her autoimmune markers including antinuclear antibody, anti-smooth muscle antibodies, anti-mitochondrial antibody, IgG levels, and anti-liver kidney microsomal 1 antibody were all negative. Serum ceruloplasmin, alpha-fetoprotein, and alpha-1 antitrypsin levels were within normal limits. A computed tomography (CT) scan of the chest, abdomen, and pelvis was performed that showed persistent airspace opacities throughout both lungs, bilateral bronchial wall thickening, and diffuse bony (skeletal) metastatic disease. The liver was described in normal morphology and size and normal bile ducts with patent hepatic vasculature.

**Figure 1:**
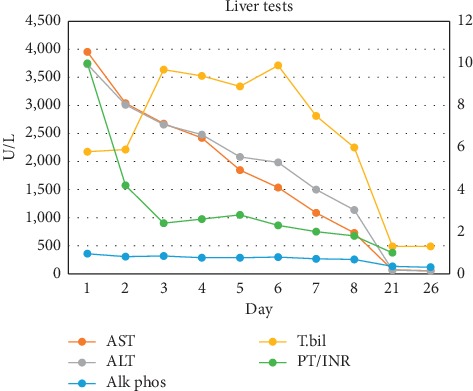
Changes in liver tests during hospitalization are plotted from admission at day 1 to day 26.

The clinical history and lab work-up were suggestive of crizotinib-associated hepatotoxicity. The patient's updated RUCAM (Roussel Uclaf Causality Assessment Method) score came out to be 8 points, which is consistent with probable drug-induced liver injury. The crizotinib was discontinued, and she was started on intravenous (IV) N-acetylcysteine (NAC) continuous infusion (100 mg/kg). She was given vitamin K 10 mg daily for three days for coagulopathy. Her liver chemistry continued to downtrend with discontinuation of crizotinib and with continuous NAC infusion for a total of 7 days. Liver biopsy was not obtained due to significant improvement of liver function tests on discontinuation of crizotinib along with intravenous NAC. The patient was discharged after eight days. Her liver test came back within normal limits 26 days after the presentation (Figure1).

## 3. Discussion

Crizotinib is a multitarget tyrosine kinase inhibitor of ALK, MET, and ROS1 [4]. Food and Drug Administration (FDA) has approved crizotinib for the treatment of patients with metastatic NSCLC whose tumors are ALK or ROS1-positive [3–5]. This report describes a case of crizotinib-induced fulminant hepatitis with recovery. Other potential causes of liver failure such as viral hepatitis, hepatic metastasis, alcoholic liver disease, or other drug-induced liver injury were excluded.

Crizotinib has been known to usually cause mild elevations in liver function tests although the exact mechanism is poorly understood [7]. The drug is metabolized in the liver by CYP3A4, and it is thought that liver injury may be due to the accumulation of toxic metabolites or immune-related mechanism [7]. The symptoms of drug-induced hepatitis are generally nonspecific; thus, the diagnosis is often delayed [7].

Hepatotoxicity was a common adverse event in patients using crizotinib in phase III studies [3, 6]. Grade 3-4 transaminase elevations occurred within the first two months of crizotinib treatment, but very rarely was there jaundice or elevations in the bilirubin and alkaline phosphatase. In the majority of patients, transaminases elevations were reversible with medication interruption or reinitiation with a lower dose and rarely required permanent discontinuation [3, 6]. Most patients, who do develop drug-induced liver injury secondary to crizotinib, are asymptomatic. However, among all the patients treated with crizotinib across clinical trials, 0.1% of patients died from acute liver failure [5]. According to prescribing information on crizotinib, monitoring of liver function test every two weeks during the first two months of treatment, then once a month, and as clinically indicated is recommended [5]. Our patient was closely monitored, and crizotinib dose was reduced to 200 mg twice daily as per recommendations. Also, she was completely asymptomatic until six weeks after the initiation of treatment with crizotinib. The dose-independent and sporadic nature of hepatotoxicity is highly suggestive of an idiosyncratic mechanism [8]. Thus, monitoring of the liver function test as per manufacturer recommendations is not sufficient to prevent idiosyncratic crizotinib-induced fulminant hepatotoxicity. The specific risk factors for crizotinib-induced hepatotoxicity are not well known. Sato et al. [9] described several factors associated with an increased risk of crizotinib-induced severe hepatotoxicity. These include CYP3A inducers or inhibitors, a history of chronic hepatitis C, collagen disorders, and antidiabetic drugs. In a retrospective study, Jung et al. [10] found that concomitant use of H2-antagonist and the presence of chronic liver disease were the factors associated with increased risk of crizotinib-induced hepatotoxicity.

On review of literature, there are only 12 cases of crizotinib-induced hepatotoxicity reported (Table 1) [7–9, 11–18]. Of these 12 patients, 8 died due to progressive liver failure in spite of discontinuation of the drug. The patients were treated with supportive treatments, lactulose and rifaximin for HE, mannitol for cerebral edema, broad-spectrum antibiotics, steroids, and in one instance plasma exchange. 2 out of the 8 patients received NAC; however, it was unclear timing and dose of NAC therapy. 4 patients recovered with resolution of hepatitis, and 3 of the patients improved with just the cessation of the medication. Our patient received NAC upon admission and for 7 days with subsequent improvement in her LFTs.

NAC is a thiol-containing agent that prevents hepatic damage by scavenging free oxygen radicals and replenishing glutathione stores [19, 20]. It has anti-inflammatory, antioxidant, inotropic, and vasodilating properties that may help limit the toxicities from crizotinib [21, 22]. The literature is not very clear about the benefit of using NAC in non-acetaminophen-induced acute liver failure (NAI-ALF). However, it has been suggested that there is benefit when administered early in the course of DILI or at the onset of hepatic encephalopathy [23]. We recommend that NAC should be considered in any patient that presents with suspected crizotinib-induced liver failure/hepatitis. NAC works as a glutathione precursor, and it has anti-inflammatory and antioxidant properties that may be helpful in limiting the toxic metabolite that results from crizotinib toxicity. NAC has a very low side-effect profile, and the potential benefit of the medication exceeds the risk.

## 4. Conclusions

This case highlights the potentially lethal side effect of crizotinib. Hopefully, postmarketing monitoring and data analysis will help in identifying the risk factors for DILI with this agent. Early use of NAC seems to be beneficial and should be considered.

## Figures and Tables

**Table 1:** Summary of patients with crizotinib-induced hepatotoxicity, characteristics, presentation, pattern of liver injury, and clinical outcome.

Year	Author	Number of cases	Age	Sex	Dosage	Duration of crizotinib exposure	Presenting symptoms	Pattern of liver injury	Mortality	Treatment	RUCAM score
2013	Ripault et al. [11]	1	69	F	500 mg qday	60 days	Fatigue	Hepatocellular	0	Withdrawal of medication with resolution	9
2014	Sato et al. [9]	1	54	F	400 mg qday	29 days	Epigastric pain	Hepatocellular	1	Withdrawal of the medication, steroids, plasma exchange	8
2016	Sassier et al. [12]	2	30, 62	M, F	250 mg/day	10 days, 30 days	None	Hepatocellular	0	Withdrawal of medication with resolution	7, 8
2016	Adhikari et al. [8]	1	56	M	250 mg BID	39 days	Fatigue, nausea/vomiting	Hepatocellular	1	NAC, mannitol, withdrawal of the medication, lactulose/rifaximin/LOLA/abx	9
2016	Van Geel et al. [7]	1	62	F	250 mg BID	24 days	Confusion	Hepatocellular	1	Supportive, withdrawal of the medication	8
2017	Brown et al. [13]	1	56	F	250 mg BID	6 weeks	Fatigue, weakness, darkening urine	Hepatocellular	1	NAC, withdrawal of the medication	NA
2017	Yasuda et al. [14]	1	51	F	250 mg BID	16 days	Abdominal pain, nausea, diarrhea	Hepatocellular	1	Steroids, supportive, withdrawal of the medication	6
2018	Charville et al. [15]	1	26	F	500 mg qday	10 weeks	Abdominal pain, jaundice	Hepatocellular	0	Withdrawal of medication with resolution, N-acetylcysteine	8
2018	Kesler et al. [16]	1	69	F	250 mg BID	4 weeks	Weakness, lightheadedness and hypotension	Hepatocellular	1	Withdrawal of the medication, lactulose/rifaximin/antibiotics	NA
2019	Zhang et al. [17]	1	37	M	250 mg BID	55 days	Dyspnea, abdominal pain	Hepatocellular	1	Withdrawal of the medication, lactulose/rifaximin/antibiotics	8
2019	Ota et al. [18]	1	66	F	250 mg BID	23 days	Fever, fatigue	Mixed	0	Withdrawal of the medication with resolution	7
2019	Kreitman et al. (Present case)	1	46	F	200 mg BID	8 weeks	Fatigue, abdominal pain, jaundice	Hepatocellular	0	Withdrawal of the medication, NAC	8

M, male; F, female; NA, not applicable (cannot be calculated).
